# Acute fibrinous and organizing pneumonia induced by cat scratch disease: a first case report

**DOI:** 10.3389/fmed.2025.1611133

**Published:** 2025-08-11

**Authors:** Zhen Yang, Zhenhua Li, Shuang Geng, Jixiang Ni, Hongling Hu

**Affiliations:** Department of Respiratory and Critical Care Medicine, The Central Hospital of Wuhan, Tongji Medical College, Huazhong University of Science and Technology, Wuhan, Hubei, China

**Keywords:** acute fibrinous and organizing pneumonia, cat scratch disease, etiology, *Bartonella henselae*, case report

## Abstract

We describe the first documented case of acute fibrinous and organizing pneumonia (AFOP) induced by cat-scratch disease (CSD). A 74-year-old female patient was admitted to the hospital with progressive dyspnea and dry cough for more than 1 month that did not respond to conventional antibiotic therapy. Physical examination revealed cat bite scars on the left index finger. Chest computed tomography (CT) revealed bilateral diffuse ground-glass opacities and consolidation. Percutaneous lung biopsy pathology confirmed the characteristic changes of AFOP, including fibrin ‘ball ‘formation in the alveolar cavity with organizing pneumonia. Polymerase chain reaction (PCR) testing was positive for *Bartonella henselae*; other pathogens and autoimmune diseases were excluded. Following treatment with doxycycline and corticosteroids, the patient exhibited clinical and radiographic improvement. This case reveals the pathogenic association between CSD and AFOP for the first time, expanding the infectious etiology spectrum of AFOP and revealing the potential association between rare infections and special types of interstitial pneumonia, providing a new perspective for differential clinical diagnoses.

## 1 Introduction

Acute fibrinous and organizing pneumonia (AFOP), first described by Beasley et al. in 2002, is a rare, acute, subacute lung injury characterized by intra-alveolar fibrin deposition and tissue pneumonia. Its etiology is unknown and may be related to various diseases and environmental exposure (1). Cat-scratch disease (CSD) is a global epidemic zoonosis caused by the gram-negative bacterium *Bartonella henselae* (*B. henselae*) that is typically characterized by benign and self-limiting regional lymphadenopathy. The incidence of lung involvement is as low as 0.15% (2). No previous cases of AFOP associated with CSD have been reported. This article is the first to report a case of AFOP caused by CSD and explore its potential mechanism, revealing the possibility of considering rare infections in atypical lung cases.

## 2 Case presentation

**Figure 1:**
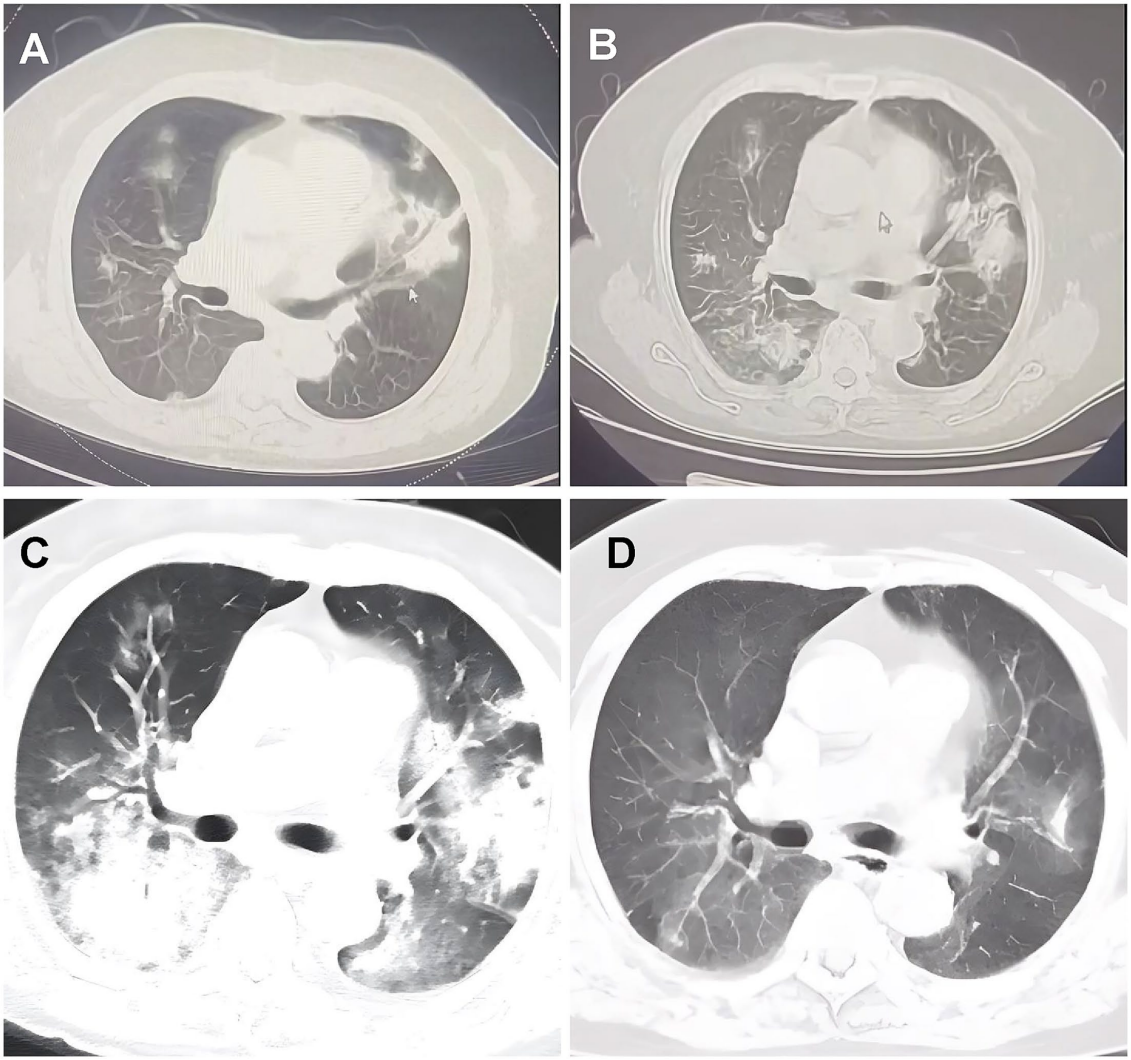
Chest computed tomography scans pictures. **(A)** September 9, 2022, CT scans from local hospitals indicated the presence of patchy shadows and solid frosted glass density lesions in both lungs. **(B)** September 16, 2022, a follow-up lung CT post anti-infective treatment showed multiple solid lesions in both lungs, with the frosted glass shadows having progressed from earlier. **(C)** September 23, 2022, she was admitted to our hospital and received an enhanced CT scan of the lungs, which indicated multiple solid lesions in both lungs and a notable increase in ground-glass shadows. **(D)** November 7, 2022, follow-up CT scans at the previous local hospital indicated considerable resorption and progress in the lung lesions.

**Figure 2:**
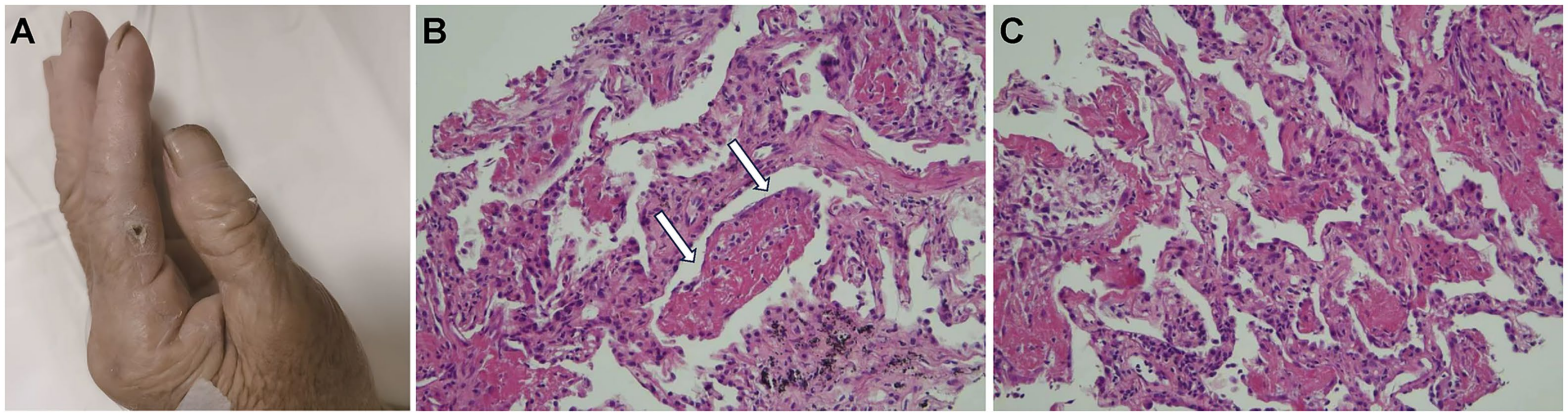
**(A)** The index finger on the patient’s left hand had a layer of charred scabs due to a cat bite from 1 month prior. **(B)** The white arrowheads indicate fibrin balls (hematoxylin and eosin staining, 20×). These fibrin balls within the alveoli lack hyaline membranes, consistent with acute fibrinous and organizing pneumonia features. **(C)** Immature fibroblasts and inflammatory cell infiltrates in the alveolar septa and within the lung tissue (hematoxylin and eosin staining, 20×).

A 74-year-old woman with dyspnea and dry cough for 1 month accompanied by intermittent fever, fatigue, loss of appetite, and night sweats was transferred to our hospital in September 2022. The patient had a history of hypertension, chronic gastritis, and iron deficiency anemia. Chest computed tomography (CT) at a local hospital showed multiple bilateral consolidations, multiple nodules, and ground-glass opacities (Figure 1A). Despite being treated with ceftriaxone and levofloxacin for a fortnight, her symptoms showed no improvement. Lung CT (Figure 1B) before hospitalization showed progression of the bilateral lung lesions. On admission, her temperature was 36.3°C; blood pressure was 119/80 mm Hg, heart rate was 94 bpm, respiratory rate was 22 breaths/min, and peripheral oxygen saturation was 93% on room air. Auscultation revealed thick breath sounds in both lungs, but no rales, heart rhythm abnormalities, or edema in either lower limb. Notably, the symptom onset occurred approximately 1 month after a cat bite on her left index finger, with a scab observed at the bite site (Figure 2A). Admission laboratory results showed a white blood cell count of 7.69 × 10^9^/L, with neutrophils at 83.1% and blood red albumin at 75 g/L; C-reactive protein level was 10.88 mg/L, and blood gas analysis showed a pH of 7.44, PO2 of 64.8 mmHg (↓), PCO2 of 34 mmHg, and SPO2 of 94% (↓). Erythrocyte sedimentation rate and serum procalcitonin levels were within normal ranges. Serological examinations for tumor markers (CEA, Cyfra 211, CA125, CA153, CA199), connective tissue disease (anti-nuclear antibody, anti-dsDNA antibody, anti-neutrophil cytoplasmic antibody, and rheumatoid factor), immunoglobulin (IgA, IgG, IgM) and human immunodeficiency virus (HIV), hepatitis, and syphilis were negative. The results for Tuberculosis T-sport, fungus type D, and blood and sputum cultures were negative. Whole-body ultrasound imaging of superficial lymph nodes revealed no swelling. Chest CT showed multiple pulmonary nodules, ground-glass opacities, a large number of patchy consolidations, and mild bilateral pleural effusions (Figure 1C).

Histological analysis of the percutaneous lung biopsy (performed on hospital day 3) for further diagnosis showed a large amount of fibrin exudate and fibrin balls in the alveolar space (Figure 2B, white arrow), a widened alveolar septum, fibrinoid exudate and fibroblast proliferation in the septum, a small amount of lymphocyte infiltration, and no obvious granulomatous inflammation changes (Figures 2B,C). Clinical symptoms and imaging features were consistent with AFOP. Special staining results were negative for GMS, gram-positivity, anti-acid, anti-acid fluorescence, and fungal fluorescence.

For etiological investigation, polymerase chain reaction (PCR) testing for *Bartonella henselae* in peripheral blood was conducted on hospital day 5, which returned positive, found no evidence of other pathogens, including rickettsia, warm-up chlamydia, Epstein–Barr virus, cytomegalovirus, toxoplasma, rubella, herpes simplex virus, mycoplasma, or chlamydia. Based on the confirmed cat exposure timeline, histological findings consistent with AFOP, and PCR confirmation of *B. henselae*, the diagnosis of AFOP secondary to cat scratch disease was established. The patient was administered oral doxycycline and intravenous corticosteroids on hospital day 6 (methylprednisolone 40 mg/d for 3 d). The cough and wheezing symptoms improved, and the dose was adjusted to 20 mg/d for 3 d. The patient was discharged 1 week after symptom improvement after regular reduction of oral methylprednisolone with outpatient follow-up. After 2 weeks of continuous oral corticosteroid administration, symptoms disappeared. After 2 months, follow-up imaging revealed that the pulmonary lesions had subsided (Figure 1D). No symptoms were present during follow-up, steroids were well tolerated, and no obvious adverse reactions were observed. No recurrence was observed during the 2-year follow-up.

## 3 Discussion

AFOP is an uncommon pathological category of idiopathic interstitial lung disease, as defined by the American Thoracic Society/European Respiratory Society in 2013 (3). It can occur in all age groups with no clear sex differences, and its clinical manifestations are nonspecific. The primary symptoms include shortness of breath, a dry cough, and fever, while other symptoms can be coughing up blood, feeling tired, experiencing chills, sweating at night, and losing weight (4). Its non-specific clinical features often lead to misdiagnosis and delayed diagnosis of AFOP. Most cases are initially diagnosed incorrectly as respiratory infections. The typical CT findings in AFOP patients are bilateral diffuse consolidation, ground-glass opacities, and multifocal parenchymal abnormalities, which are generally basal or peripheral (5).

AFOP diagnosis is based on lung biopsy pathology, characterized mainly by fibrin ‘balls’ in alveoli and patchy organizing pneumonia (1). It is marked by both acute and chronic inflammation, along with type II lung cell growth near the fibrin deposition zone, myxoid degeneration causing alveolar septum expansion, minimal alterations in the lung tissue region, and no fibrin exudation. There is no uniform treatment plan for AFOP, and antibiotics are ineffective. Glucocorticoids are the most common and effective treatment, though a consensus has not yet been reached regarding their dose and course of treatment. Individual treatment selection should be based on the patient’s condition, imaging changes, and drug reactions. Patients suffering from AFOP associated with autoimmune diseases might benefit from using immunosuppressive agents like cyclophosphamide, mycophenolate mofetil, cyclosporine, azathioprine, and tacrolimus (6). The patient was admitted to a local hospital in this case due to non-specific symptoms like fever and a dry cough. Based on chest imaging, the patient was initially diagnosed with a pulmonary infection, though empirical antibiotic treatment was ineffective after 2 weeks. After further CT-guided percutaneous lung biopsy, AFOP was diagnosed, and the patient was treated with corticosteroids with a good prognosis.

The pathogenesis of AFOP is still unclear and needs further study. According to Qnishi et al. (7), AFOP might represent an initial histological model for lung injury wound healing. The development of cellulose balls could be akin to the formation of hyaline membranes in diffuse alveolar damage. Under different conditions, the protein slurry, including fibrinogen, penetrates the alveolar cavity from the pulmonary capillaries, where it is transformed into fibrin by coagulants, resulting in the formation of fibrin balls. Researchers have speculated that AFOP may be a lung manifestation of autoimmune disorders, and the abnormal immune system during tumor and tumor-related treatment may play an important role in its pathogenesis (8). The pathological manifestation of programmed death molecule 1 antibody-associated pneumonia is AFOP, which can be fatal, further suggesting that the immune mechanism may be an important factor in the occurrence of AFOP, which should be considered by clinicians (9). The etiology of AFOP is complex and diverse, as it may arise without a known cause or be secondary to other conditions. It can be triggered by numerous factors such as autoimmune disorders, medications, cancers, and environmental factors (10, 11). These factors were not observed in the present case. Furthermore, it may also develop secondary to a range of infections. Pathogens including viruses (H1N1 influenza, Epstein–Barr virus, cytomegalovirus, and novel coronavirus), bacteria (*Haemophilus influenzae*, *Acinetobacter baumannii*, *Mycoplasma pneumoniae*, *Chlamydia*, and *Legionella*), and fungi (*Pneumocystis jiroveci* and *Aspergillus fumigatus*) have been reported as causative agents (12–17).

However, the aforementioned etiology was not found in this patient using traditional detection methods, though *B. henselae* was detected by PCR. This case of AFOP was caused by a rare CSD infection, which further expands the understanding of AFOP etiology. The patient was bitten by a cat 1 month prior and was left with a left-hand eschar. Comprehensive diagnostic tests were performed to exclude other causes of infection; blood, urine, and throat cultures were negative, as were serum antibodies for bacterial and viral infections. Although this patient did not undergo next-generation sequencing owing to cost constraints, PCR was sufficient to support the diagnosis of CSD. This case provides an important reference for clinical practice, clarifying the causal relationship between CSD and AFOP using PCR. Additionally, this is the first known case of AFOP attributed to CSD, based on our information.

CSD, first described by Debré et al. in 1950, is a rare infection but globally epidemic zoonosis that is common in domestic cat families, caused by the gram-negative bacterium *B. henselae* (18). It usually manifests as a benign, self-limiting regional lymphadenopathy occurring 1–8 weeks after being scratched or bitten by a domestic cat. The diagnosis of CSD depends on serological testing or PCR because *B. henselae* cannot be detected by conventional blood culture (19). Although serological testing usually has high sensitivity, it cannot distinguish between ongoing and past infections. As Gaoz et al. reported in 2022, PCR has been shown to detect *B. henselae* in fresh tissue or purulent samples with high sensitivity and specificity (20).

Although clinical manifestations of CSD are usually self-limiting, blood-borne bacterial transmission to the lungs leads to lung inflammation and infiltration (18). This blood-borne transmission is facilitated by bacterial persistence within erythrocytes and endothelial cells, enabling systemic spread and pulmonary colonization (21). Pneumonia caused by CSD is rare, though pulmonary infiltration can manifest as pneumonia or interstitial lung disease. This case suggests that Bartonella infection can trigger the pathological process of alveolar fibrin deposition and tissue pneumonia. Although the mechanism of lung involvement in CSD is not yet clear, possible pathogeneses include pathogen invasion through skin damage (such as a cat scratch), entering the blood circulation through the lymphatic system, where bacteria survive in the blood and spread to distant organs, including the lungs (18). Intracellular survival is enhanced by transcriptional downregulation of invasion genes (e.g., BadA adhesin), enabling immune evasion and chronic persistence (22). *Bartonella* infection induces excessive inflammatory responses, leading to alveolar fibrin deposition and tissue pneumonia (23). Crucially, the Bartonella autotransporter BafA activates host VEGF-R2 signaling (24), driving pathological angiogenesis and vascular leakage. This disrupts the alveolar-capillary barrier, permitting fibrinogen extravasation into airspaces—a hallmark of AFOP. Concurrently, bacterial lipoproteins trigger TLR-mediated cytokine storms (TNF-*α*/IL-6), amplifying tissue damage (25).

In addition, Bartonella may directly damage alveolar epithelial and endothelial cells, leading to the destruction of the alveolar capillary barrier and further promoting fibrin exudation and tissue pneumonia (26), through secreted proteases and BafA-mediated endothelial proliferation (24), further compromising barrier integrity and promoting fibrin exudation. In immunocompromised hosts, this can escalate to hemophagocytic lymphohistiocytosis (HLH), where macrophage activation secretes fibrogenic factors (e.g., TGF-*β*) that drive organizing pneumonia (25). Margileth et al. reported 13 cases of rare pneumonia or pleural changes caused by CSD, six of which showed bilateral lung infections and three of which were treated with hormones. After treatment, two patients had a good prognosis, and only one patient died of low human immunodeficiency virus-positive immunity. AFOP may also occur in this type of severe pneumonia; however, the original case in this previous report did not include the concept of AFOP and no histopathological biopsy was performed to confirm it (27). Therefore, this case is the first to propose that CSD causes AFOP, hoping to provide clinicians with etiological experience.

## 4 Conclusion

This case reports the world ‘s first rare AFOP caused by CSD, aiming to improve our understanding, diagnosis, and treatment of AFOP, emphasizing the necessity of considering rare infections in atypical lung cases. Early diagnosis and treatment with corticosteroids can improve prognosis. AFOP should be considered in patients with pulmonary symptoms after cat bites or scratches, as it may be a rare complication of CSD.

## Data Availability

The raw data supporting the conclusions of this article will be made available by the authors, without undue reservation.
